# Chilaiditi sign mimicking pneumoperitoneum with ischemic sigmoid volvulus: CT diagnosis of a surgical emergency

**DOI:** 10.1016/j.radcr.2026.03.053

**Published:** 2026-05-09

**Authors:** Amanuel Mesfin Oljira, Sinbona Ararsa Keneni, Dessalegn Abdisa Hordofa, Boka Imiru Shoro, Rabirra Waktola Gonfa, Tesfaye Bekele Bidirat, Aemon Berhane Fissha, Dagem Asseged Gebremichael

**Affiliations:** a Department of Surgery, Ambo University College of Medicine and Health Science, Ambo, Ethiopia; b Department of Radiology, Ambo University College of Medicine and Health Science, Ambo, Ethiopia; c Department of Public Health, School of Medicine, College of Health Sciences, Addis Ababa university, Addis Ababa, Ethiopia

**Keywords:** Chilaiditi sign, Chilaiditi syndrome, Sigmoid volvulus, Pseudopneumoperitoneum, Case report

## Abstract

Chilaiditi syndrome involves the abnormal position of bowel between the liver and diaphragm, causing symptoms in the patient, which can mimic pneumoperitoneum on plain X-rays. Although sigmoid volvulus associated with Chilaiditi syndrome has been previously reported, the principal educational value of the present case lies in demonstrating how contrast-enhanced computed tomography (CT) can simultaneously distinguish pseudopneumoperitoneum from true pneumoperitoneum and identify ischemic volvulus requiring urgent surgical management. We describe a 45-year-old man who came to the hospital with acute large-bowel obstruction and visible gas under the right diaphragm. An upright X-ray showed subdiaphragmatic gas along with haustral markings. A contrast-enhanced CT scan confirmed the Chilaiditi sign and showed a sigmoid volvulus with mesenteric whirling and reduced enhancement, indicating ischemia. Subtle peripheral hepatic enhancement was also noted, likely reflecting adjacent inflammatory change. Emergency surgery found a gangrenous 720° sigmoid volvulus trapped between the liver and the diaphragm; a sigmoid colectomy and Hartmann’s procedure were performed, followed by a successful colostomy reversal. The patient remained well at 12-month follow-up. This case highlights how CT can help distinguish between pseudopneumoperitoneum and true pneumoperitoneum while also identifying ischemic volvulus that needs urgent surgery. The case further emphasizes the value of CT in directing timely operative management in patients with apparent subdiaphragmatic free air and suspected bowel ischemia.

## Introduction

The Chilaiditi sign refers to the interposition of bowel, most commonly colon, between the liver and the right hemidiaphragm. When it is associated with symptoms like abdominal pain, distension, and obstruction, it is called Chilaiditi syndrome [1,2]. It can be confused with pneumoperitoneum on a plain radiograph, posing a significant problem in diagnosis, which in turn can affect management.

Sigmoid volvulus is a significant cause of large bowel obstruction in the ‘volvulus belt,’ including Ethiopia, where delayed presentation increases the likelihood of ischemia and gangrene [3,4]. Although Chilaiditi syndrome associated with sigmoid volvulus has been previously reported, the key educational value of the present case is not the coexistence alone, but the radiologic demonstration of how contrast-enhanced CT resolves the differential diagnosis of apparent subdiaphragmatic free air while simultaneously identifying ischemic volvulus requiring urgent surgical management [[5], [6], [7]]. Reporting radiologic case reports using structured guidance can improve completeness and transparency, particularly for imaging findings and diagnostic reasoning [8]. Computed tomography is a valuable imaging tool in diagnosing Chilaiditi syndrome, including cases of interposition of colon in the absence of free air in the peritoneum. It is useful in diagnosing the ischemic features of volvulus, such as mesenteric whirl, and is essential in planning appropriate management, including surgery [9]. We present a case of gangrenous sigmoid volvulus with Chilaiditi sign in which computed tomography was pivotal both in distinguishing pseudopneumoperitoneum from true pneumoperitoneum and in demonstrating imaging features of bowel ischemia.

## Case report

A 45-year-old Ethiopian man came to the emergency department with 3 days of colicky abdominal pain, increasing abdominal swelling, repeated vomiting, loss of appetite, and constipation. He had no prior abdominal surgery, no chronic medical illness, and no similar previous episodes. He had no history of chronic constipation, laxative use, and followed a high-fiber diet. He had no psychiatric or neurological issues, did not stay in a care facility, and took no opioids or anticholinergic medications. He was fully independent without mobility issues.

During the examination, he appeared uncomfortable and slightly dehydrated, with dry mouth; his capillary refill was normal (2 seconds). His vital signs were: blood pressure 134/80 mmHg, pulse rate 103 beats/minute, respiratory rate 24 breaths/minute, and temperature 38.1°C. His abdomen was notably distended and hypertympanic on percussion, with diffuse guarding and generalized direct and rebound tenderness across all quadrants. Bowel sounds were hypoactive. A digital rectal exam revealed an empty rectum, and the hernia sites were free.

Laboratory tests showed WBC 13,000/µL, hemoglobin 13.2 g/dL, platelets 251,000/µL, CRP 32 mg/dL, and serum lactate 4.2 mmol/L. Electrolytes, kidney, and liver function tests were normal. An arterial blood gas test was not done.

The main initial differential diagnoses included perforated peptic ulcer, sigmoid volvulus, cecal volvulus, distal colonic obstruction with proximal blowout, and Chilaiditi syndrome.

**Fig. 1:**
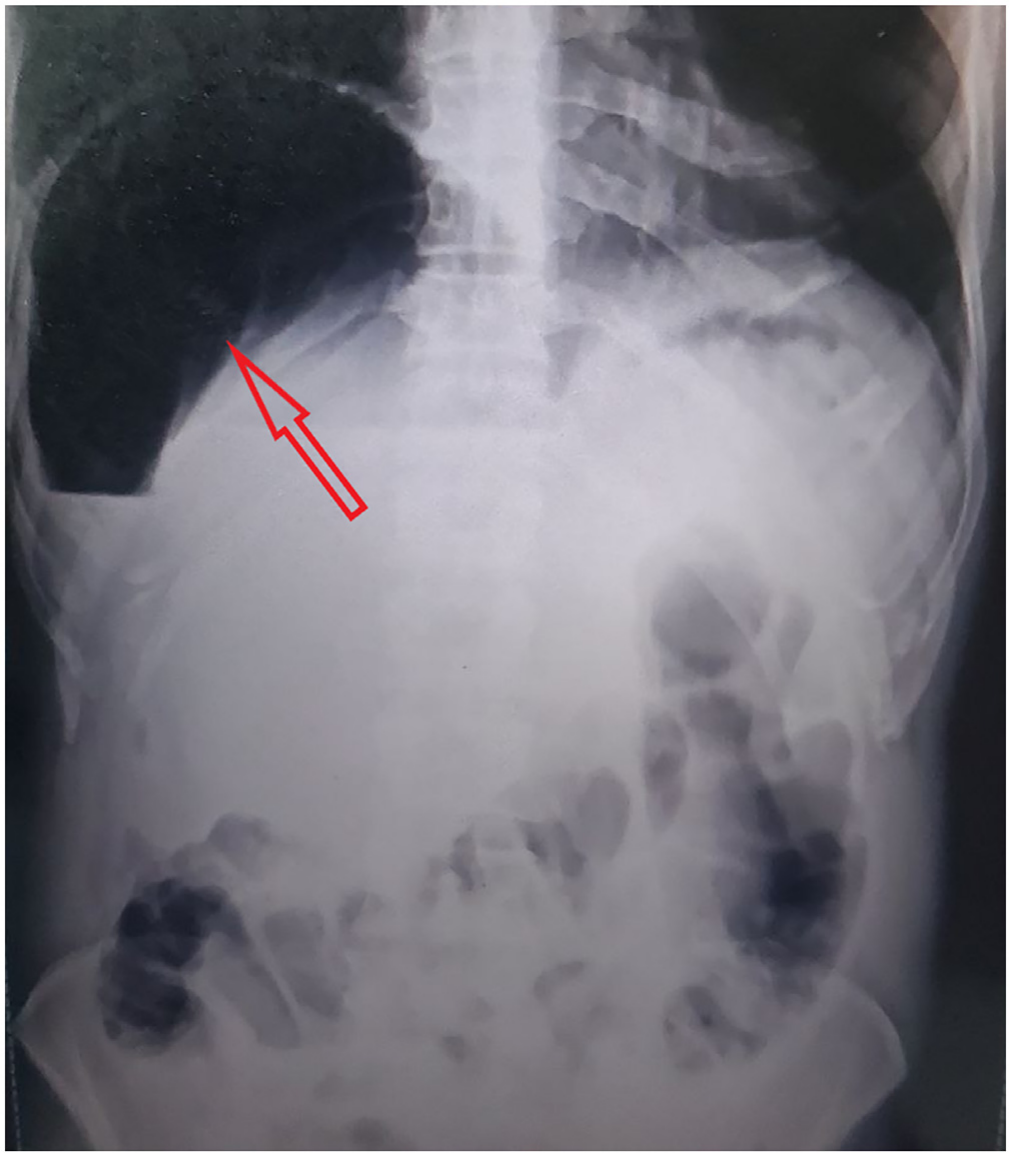
Erect anteroposterior abdominal radiograph demonstrating the interposition of a gas-distended colonic segment between the liver and the right hemidiaphragm, characterized by the presence of visible haustral markings within the subphrenic lucency, and significant gaseous dilatation of the proximal colonic loops is noted.

An upright abdominal X-ray showed gas under the right diaphragm with visible haustral markings, suggesting colonic interposition rather than free air in the abdomen [1,2] [Fig. 1]. Due to concerns about obstruction and subdiaphragmatic gas, a contrast-enhanced abdominal CT was done.

**Fig. 2:**
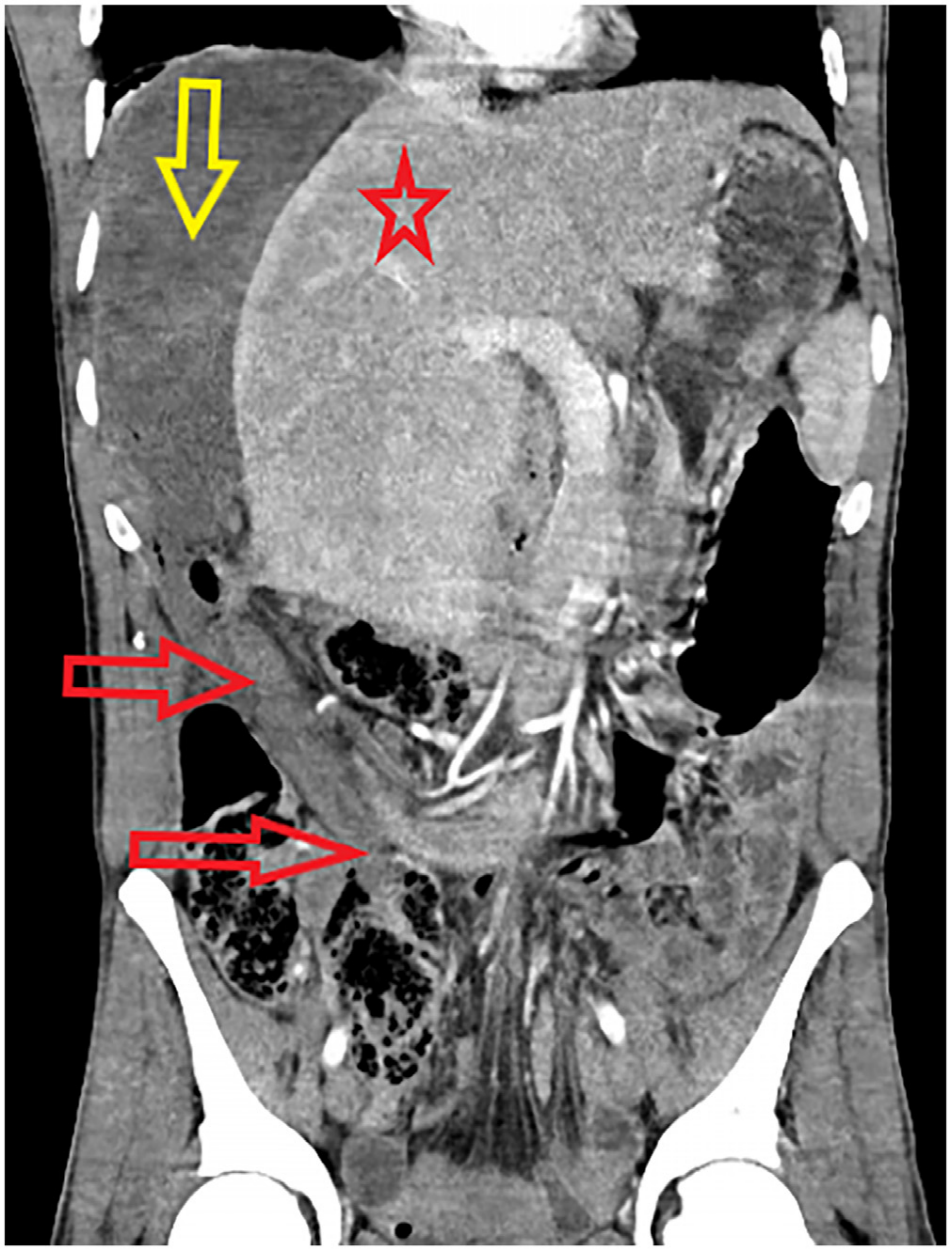
Axial contrast-enhanced CT image demonstrating a massively gas-distended colonic loop (red arrow) interposed between the liver (red star) and the right lateral abdominal wall. Multiple yellow arrows identify a curvilinear band of peripheral hepatic enhancement.

**Fig. 3:**
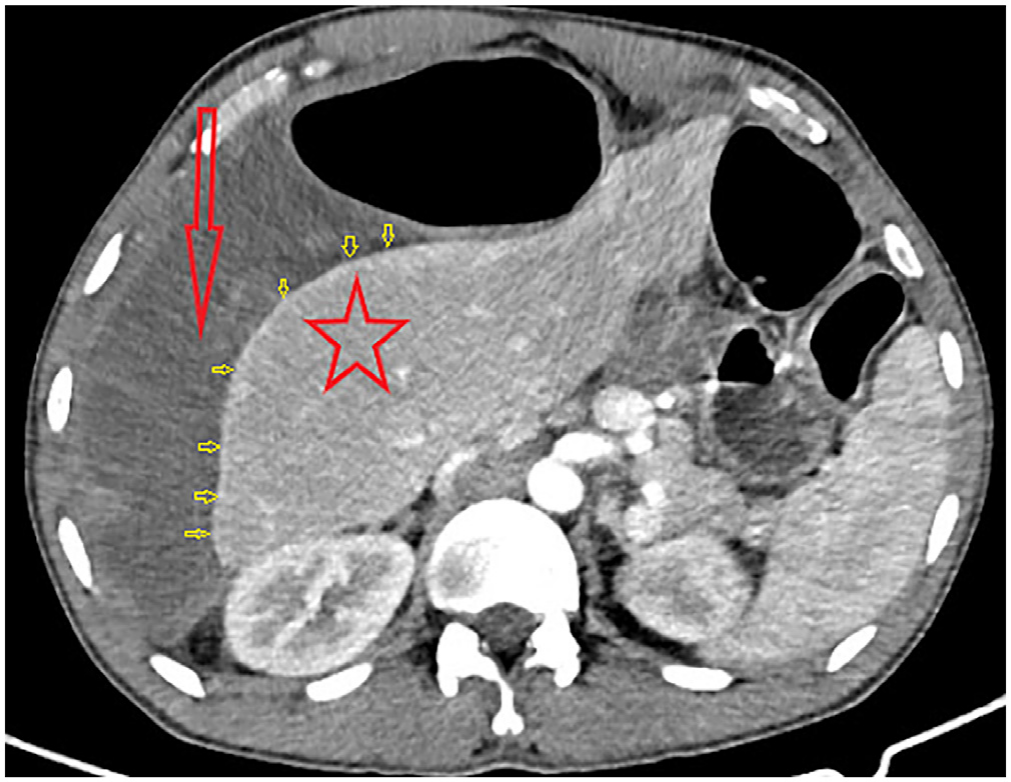
Coronal CECT image illustrating the superior migration of the dilated colon into the right subphrenic space (yellow arrow) and the inferiorly displaced liver (red star); the inferior mesenteric whirl sign (red arrows) identifies the transition point and site of torsion**.**

**Fig. 4:**
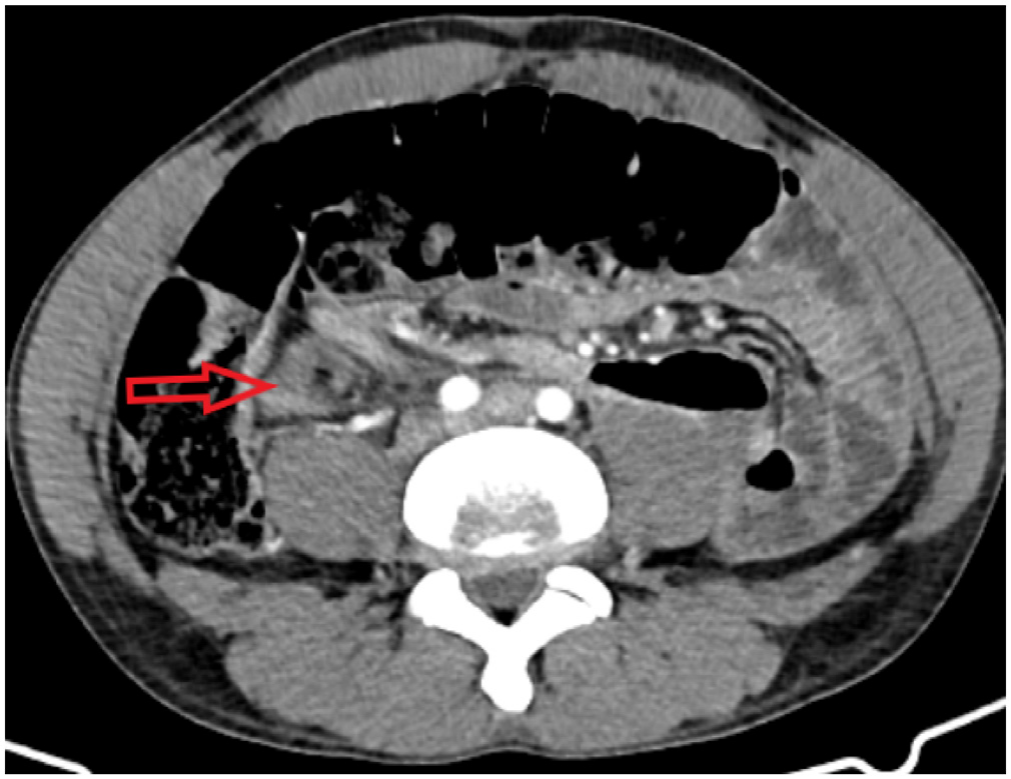
Axial CECT image at the level of the lower abdomen providing a definitive view of the pathognomonic whirl sign (red arrow), characterized by the twisting of the sigmoid mesentery and its associated vasculature, confirming acute sigmoid volvulus as the etiology of the Chilaiditi syndrome.

The contrast-enhanced CT scan of the abdomen demonstrated markedly dilated colonic loops in the right upper abdomen, with dilated sigmoid loops interposed between the liver and the right hemidiaphragm, displacing the liver leftward and confirming the Chilaiditi sign [Fig. 2, Fig. 3, Fig. 4]. Post-contrast axial and coronal images showed whirling of the sigmoid mesentery extending from the pelvis to the right upper abdomen, while the wall of the dilated sigmoid colon was thinned with reduced enhancement, indicating ischemia [Fig. 3]. There was no free intraperitoneal air. Subtle peripheral hepatic enhancement was also present, likely reflecting adjacent inflammatory change [Fig. 2].

**Fig. 5:**
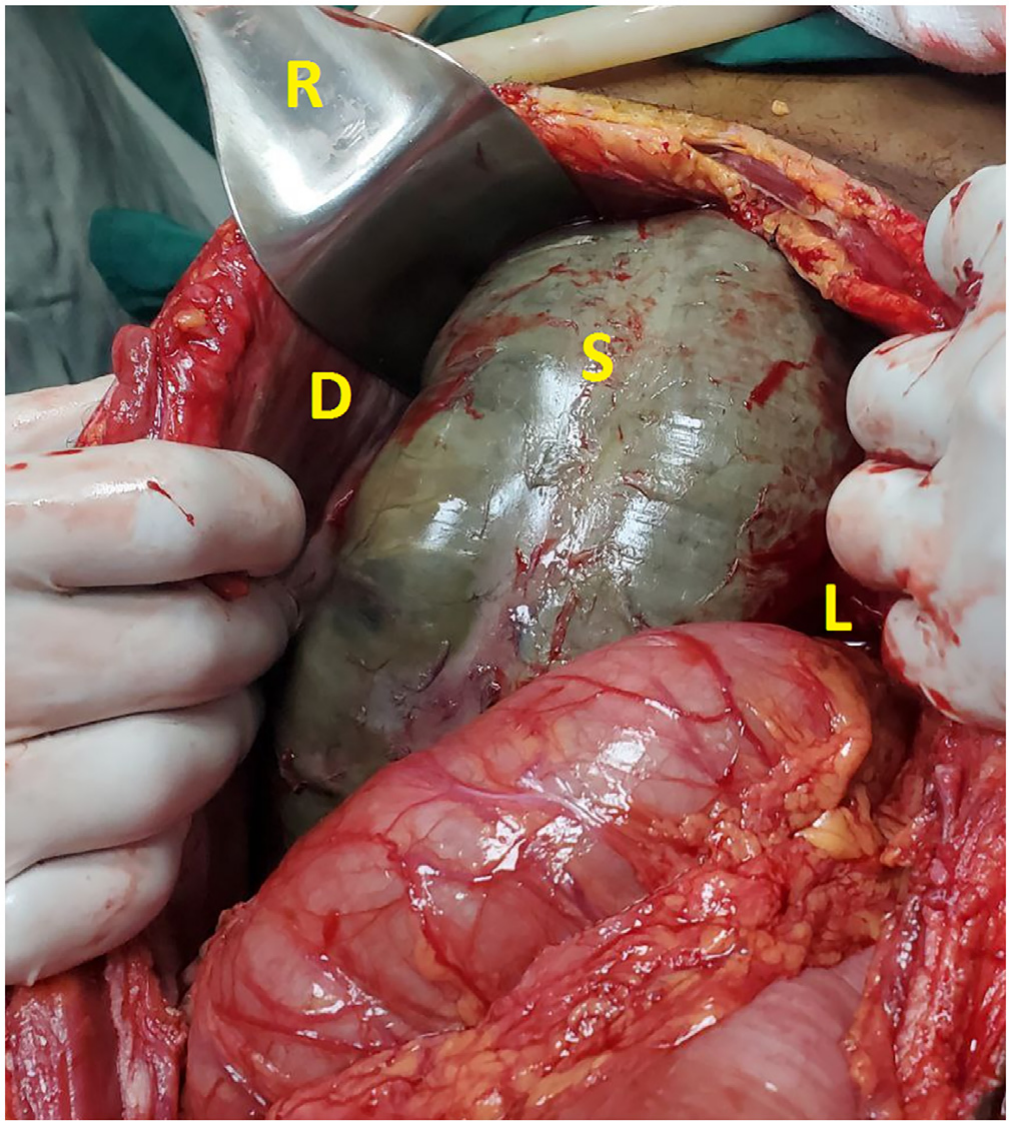
An intraoperative photograph during exploratory laparotomy illustrating the surgical retractor (R) used to elevate the abdominal surface of the right diaphragm (D), revealing the massively distended and gangrenous sigmoid colon (S) occupying the right subdiaphragmatic space. The necrotic sigmoid loop (S) is situated superior to the liver (L), which has been displaced inferiorly.

**Fig. 6:**
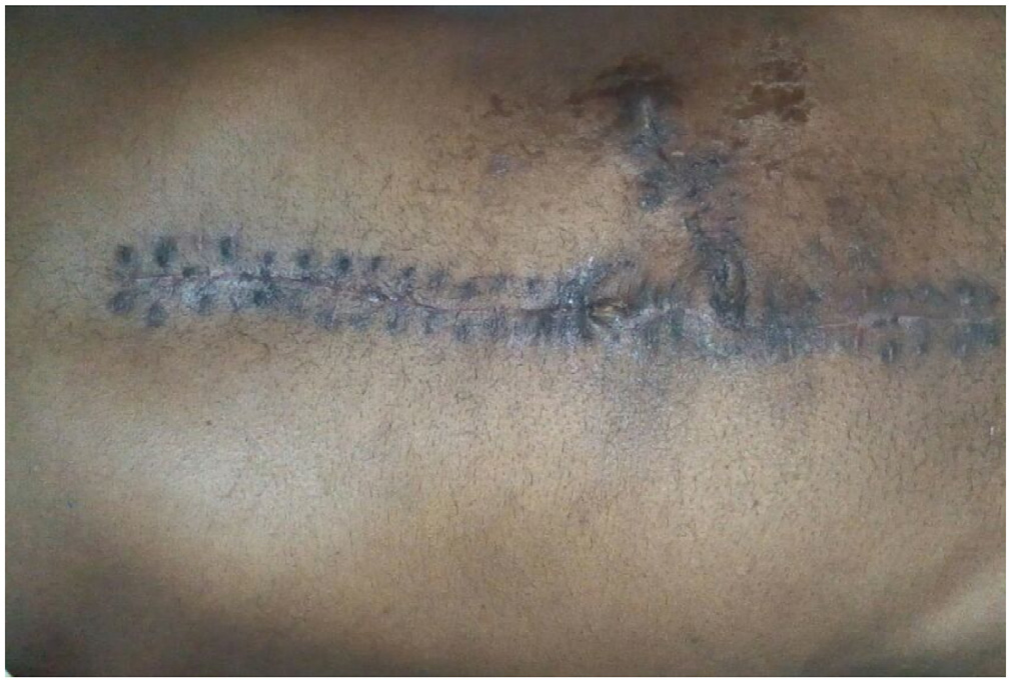
Six months post colostomy reversal picture showing healed abdominal wound.

The patient received intravenous fluids as per protocol, had a nasogastric tube for decompression, and was given intravenous ceftriaxone (1 gram) and metronidazole (500 mg) prior to skin incision. Given the suspected ischemia based on the CT findings, clinical features, and laboratory workup—and the lack of endoscopy services at our hospital—endoscopic detorsion was not attempted. The time from assessment in the emergency department to the operating room was about 2 hours (Figs. 5 and 6).

During the emergency exploratory laparotomy through a midline incision, about 100 mL of fluid was found in the peritoneal cavity. A redundant sigmoid colon was twisted 720° counterclockwise and showed signs of gangrene, lying between the liver and diaphragm. The proximal colon was grossly normal, with no megacolon, and there were no perforations or fecal contamination. A 40-cm gangrenous segment of the sigmoid was removed en bloc without untwisting; both proximal and distal bowel ends were healthy. Hartmann’s procedure was done with an end colostomy, and the rectal stump was closed and labeled. Histopathology was not available at our setup.

The postoperative recovery was smooth. The patient was discharged on the fourth day after initial surgery, following education on how to care for the stoma. He did not experience any wound infections, stoma problems, or readmission. Colostomy reversal with a tension-free end-to-end anastomosis was done 3 months later. At 12-month follow-up, he had normal bowel function and no evidence of recurrence. A timeline is provided in Table 1.

**Table 1:** Timeline of events.

Time point	Event
Day −3 to day 0	Onset of colicky abdominal pain with progressive distension, repeated vomiting, anorexia, and obstipation (3-day duration before presentation).
Day 0 (ED presentation)	Vitals: BP 134/80 mmHg, pulse 103/min, RR 24/min, temperature 38.1°C. Exam: hyper-tympanic distended abdomen with diffuse guarding and generalized rebound tenderness; hypoactive bowel sounds; DRE: empty rectum; hernial orifices free.
Day 0 (initial labs)	WBC 13,000/µL; CRP 32 mg/dL; serum lactate 4.2 mmol/L; Hb 13.2 g/dL; platelets 251,000/µL; electrolytes/renal/liver function tests within normal limits; ABG not performed.
Day 0 (initial imaging)	Upright abdominal radiograph: right subdiaphragmatic gas with visible haustral folds suggesting colonic interposition rather than free intraperitoneal air.
Day 0 (CT imaging)	Contrast-enhanced abdominal CT: dilated sigmoid loops interposed between liver and right hemidiaphragm with leftward liver displacement (Chilaiditi sign); mesenteric whirl extending pelvis → right upper abdomen; thinned sigmoid wall with decreased post-contrast enhancement and pneumatosis consistent with ischemic sigmoid volvulus; no free intraperitoneal air.
Day 0 (initial management)	Fluid resuscitation per protocol; NG decompression; antibiotics: ceftriaxone 1 g IV BID + metronidazole 500 mg IV TID; endoscopic detorsion not attempted (suspected ischemia + peritoneal signs + endoscopy service unavailable).
Day 0 (time to OR)	Transfer from ED assessment to the operating room in ∼ 2 h.
Day 0 (operative findings)	Midline laparotomy: ∼ 100 mL reactive peritoneal fluid; redundant sigmoid colon twisted 720° counterclockwise and gangrenous; proximal colon normal (no megacolon); no perforation; no fecal contamination.
Day 0 (procedure)	En bloc resection of 40 cm gangrenous sigmoid colon without detorsion; proximal and distal ends viable; Hartmann’s procedure with end colostomy; rectal stump closed and tagged; histopathology not available.
Postoperative day 4	Discharged after stoma education; no wound infection, no stoma complications, no readmission.
3 mo	Colostomy reversal with tension-free end-to-end colorectal anastomosis.
12 mo	Follow-up: normal bowel function; no recurrence.

## Discussion

The presence of sub-diaphragmatic free gas in acute abdomen and obstruction symptoms poses an important differential diagnosis, including true pneumoperitoneum due to a perforated viscus, diaphragmatic hernia, subphrenic abscess, cecal volvulus, and toxic megacolon. Chilaiditi syndrome is one of the important mimics of pseudopneumoperitoneum and was considered in the present case based on the presence of haustral markings on the plain abdominal radiograph, and confirmed on contrast-enhanced CT scan showing the interposed colon in the absence of free intraperitoneal air, thereby avoiding inappropriate management [1,2].

Diagnostic pitfalls and misinterpretation of Chilaiditi sign as true pneumoperitoneum have been documented in the literature, underscoring the importance of CT when plain radiographic findings are equivocal [10].

In the present case, the subtle peripheral hepatic enhancement on contrast-enhanced CT was interpreted as a supportive but nonspecific finding, likely reflecting reactive perihepatic inflammatory change adjacent to the gangrenous sigmoid colon. Hepatic capsular or perihepatic enhancement has been described as a radiologic sign of localized peritoneal inflammation/perihepatitis, and its presence in our patient is compatible with adjacent inflammatory change rather than a primary hepatobiliary process [11].

Colonic interposition may coexist with colonic volvulus due to increased mobility and redundancy. However, the principal educational value of this case is not the coexistence of sigmoid volvulus and Chilaiditi syndrome alone, since this association has been previously reported, including cases of sigmoid volvulus, recurrent volvulus, and viable bowel amenable to non-operative or elective management [[5], [6], [7]].

Additional reports have also described Chilaiditi syndrome associated with volvulus involving other colonic segments, particularly transverse colon volvulus, supporting the concept that colonic redundancy and abnormal mobility may predispose to multiple volvulus patterns in this anatomic setting [12,13].

What distinguishes the present case is the CT-based demonstration of 2 clinically decisive findings at the same time: (1) pseudopneumoperitoneum due to colonic interposition rather than free intraperitoneal air, and (2) imaging evidence of ischemic sigmoid volvulus requiring urgent surgery.

The present recommendations suggest endoscopic detorsion of the sigmoid colon in uncomplicated sigmoid volvulus, but urgent surgical resection may be necessary in cases of suspected ischemia and perforation [13,14]. In our patient, the reduced mural enhancement, mesenteric whirling, peripheral inflammatory change, fever, generalized peritoneal signs, leukocytosis, elevated CRP, and raised lactate all supported immediate operative intervention rather than attempted endoscopic detorsion [14,15].

Hartmann’s procedure was selected as a safe staged option because the sigmoid colon was gangrenous and local resource constraints increased concern for anastomotic risk in the emergency setting.

Ethiopia falls within the “volvulus belt,” where sigmoid volvulus is a common cause of intestinal obstruction, and delayed presentation increases the risk of ischemic complications [3,4,9]. Recent local case literature also highlights that Chilaiditi syndrome is rare and prone to diagnostic confusion, reinforcing the importance of careful radiographic interpretation and CT when available [16].

## Limitations

Histopathology and arterial blood gas tests were not performed; however, imaging, surgical findings, lab results, and clinical progression supported the diagnosis and severity.

## Conclusion

Chilaiditi syndrome should be considered in patients with bowel obstruction symptoms and a notable right subdiaphragmatic air shadow on radiograph. Contrast-enhanced CT is crucial for confirming pseudopneumoperitoneum (interposed bowel without free air) and identifying ischemic features that require urgent surgical intervention. In the present case, the main educational value lies in the CT-based differentiation of apparent pneumoperitoneum from Chilaiditi-related pseudopneumoperitoneum together with simultaneous demonstration of ischemic sigmoid volvulus, directly guiding emergency surgical management.

## Patient perspective

``Before the surgery, the pain was a solid 10—I couldn't stop vomiting and felt like I was bursting. Waking up, I was sore and had a few new scars, but that agonizing pressure was finally gone. The first few days of just walking the halls and waiting for my system to `restart' were tough, but eating my first real meal without pain felt like a miracle.''

## Ethics statement

Ethical approval was not required for this single anonymized case report in accordance with local institutional policy.

## Data availability

The data that support the findings of this study are available from the corresponding author upon reasonable request.

## Declaration of the use of generative AI and AI-assisted technologies

During the preparation of this work, the authors did not use generative AI and AI-assisted technologies.

## Patient consent

Written informed consent was obtained from the patient to publish this case report and accompanying images. The signed consent form is kept by the authors and can be provided upon request.

## Appendix Supplementary materials

Image, application 1
