# Supplementary material for: Chilaiditi sign mimicking pneumoperitoneum with ischemic sigmoid volvulus: CT diagnosis of a surgical emergency
Source: Radiol Case Rep. 2026 May 9;21(8):3145–50. doi: 10.1016/j.radcr.2026.03.053 (PMC13181245; doi:10.1016/j.radcr.2026.03.053)
Supplement: Supplementary file 1 [file mmc1.pdf]

CARE Checklist of information to include when writing a case report

CARE case report guidelines

(cc) BY-NC-ND

Topic	Item	Checklist item description	Reported on Line
Title	1	The diagnosis or intervention of primary focus followed by the words "case report"	1-2
Key Words	2	2 to 5 key words that identify diagnoses or interventions in this case report, including "case report"	16-17
Abstract (no references)	3a	Introduction: What is unique about this case and what does it add to the scientific literature?	4-6
	3b	Main symptoms and/or important clinical findings	7-9
	3c	The main diagnoses, therapeutic interventions, and outcomes	11-14
	3d	Conclusion—What is the main "take-away" lesson(s) from this case?	15-17
Introduction	4	One or two paragraphs summarizing why this case is unique (**may include references**)	20-36
Patient Information	5a	De-identified patient specific information.	38
	5b	Primary concerns and symptoms of the patient.	39-43
	5c	Medical, family, and psycho-social history including relevant genetic information	40-43
	5d	Relevant past interventions with outcomes	none
Clinical Findings	6	Describe significant physical examination (PE) and important clinical findings.	44-50
Timeline	7	Historical and current information from this episode of care organized as a timeline	86
Diagnostic Assessment	8a	Diagnostic testing (such as PE, laboratory testing, imaging, surveys).	58-65
	8b	Diagnostic challenges (such as access to testing, financial, or cultural)	79
	8c	Diagnosis (including other diagnoses considered)	54-56
	8d	Prognosis (such as staging in oncology) where applicable	not applicable
Therapeutic Intervention	9a	Types of therapeutic intervention (such as pharmacologic, surgical, preventive, self-care)	69-89
	9b	Administration of therapeutic intervention (such as dosage, strength, duration)	69
	9c	Changes in therapeutic intervention (with rationale)	none
Follow-up and Outcomes	10a	Clinician and patient-assessed outcomes (if available)	83-89
	10b	Important follow-up diagnostic and other test results	83-89
	10c	Intervention adherence and tolerability (How was this assessed?)	83-89
	10d	Adverse and unanticipated events	none
Discussion	11a	A scientific discussion of the strengths AND limitations associated with this case report	90-117
	11b	Discussion of the relevant medical literature **with references**.	100-105
	11c	The scientific rationale for any conclusions (including assessment of possible causes)	106-112
	11d	The primary "take-away" lessons of this case report (without references) in a one paragraph conclusion	121-130
Patient Perspective	12	The patient should share their perspective in one to two paragraphs on the treatment(s) they received	126-130
Informed Consent	13	Did the patient give informed consent? Please provide if requested	Yes ☒ No ☐
